# Recent progress of prognostic biomarkers and risk scoring systems in chronic lymphocytic leukemia

**DOI:** 10.1186/s40364-020-00222-3

**Published:** 2020-09-07

**Authors:** Xiaoya Yun, Ya Zhang, Xin Wang

**Affiliations:** 1 Department of Hematology, Shandong Provincial Hospital, Cheeloo College of Medicine, Shandong University, Jinan, 250021 Shandong China; 2 Department of Hematology, Shandong Provincial Hospital Affiliated to Shandong First Medical University, No.324, Jingwu Road, Jinan, 250021 Shandong China; 3 School of Medicine, Shandong University, Jinan, 250012 Shandong China; 4 Shandong Provincial Engineering Research Center of Lymphoma, Jinan, 250021 Shandong China; 5 National clinical research center for hematologic diseases, Jinan, 250021 Shandong China

**Keywords:** Chronic lymphocytic leukemia, Prognosis, Prognostic biomarkers, Risk scoring systems

## Abstract

Chronic lymphocytic leukemia (CLL) is the most prevalent adult leukemia with high heterogeneity in the western world. Thus, investigators identified a number of prognostic biomarkers and scoring systems to guide treatment decisions and validated them in the context of immunochemotherapy. A better understanding of prognostic biomarkers, including serum markers, flow cytometry outcomes, IGHV mutation status, microRNAs, chromosome aberrations and gene mutations, have contributed to prognosis in CLL. Del17p/ TP53 mutation, NOTCH1 mutation, CD49d, IGHV mutation status, complex karyotypes and microRNAs were reported to be of predictive values to guide clinical decisions. Based on the biomarkers above, classic prognostic models, such as the Rai and Binet staging systems, MDACC nomogram, GCLLSG model and CLL-IPI, were developed to improve risk stratification and tailor treatment intensity. Considering the presence of novel agents, many investigators validated the conventional prognostic biomarkers in the setting of novel agents and only TP53 mutation status/del 17p and CD49d expression were reported to be of prognostic value. Whether other prognostic indicators and models can be used in the context of novel agents, further studies are required.

## Background

Chronic lymphocytic leukemia (CLL) is the most prevalent adult leukemia in the western world. The disease typically occurs in older patients and presents a variable disease course. The diagnosis of CLL requires the presence of more than 5 × 10^9^/L B lymphocytes in the peripheral blood, sustained at least 3 months. The leukemia cells found by blood smear are characteristically small, mature lymphocytes with a narrow border of cytoplasm and a dense nucleus lacking discernible nucleoli and having partially aggregated chromatin. CLL cells coexpress the surface antigen CD5 together with the B-cell antigens CD19, CD20 and CD23 [1]. More than 15,000 newly diagnosed cases and 4500 deaths are currently estimated in the United States [2]. However, only patients with advanced, active and symptomatic disease need therapy. Novel agents, including inhibitors of B-cell receptor signaling pathway (ibrutinib, acalabrutinib, idelalisib and duvelisib) and the inhibitor of the anti-apoptotic protein BCL-2 (venetoclax), are superior compared to conventional chemoimmunotherapy (CIT) regimens. Cellular immunotherapy with chimeric antigen receptor T-cell (CAR-T) and allogeneic stem cell transplant (allo-SCT) are available for high-risk patients [3–9]. New challenges emerge when patients relapse on novel agents, and optimal sequencing strategies have not been established. Current clinical trials aim to deeper remissions and long-term control of CLL [2].

Due to the high heterogeneity of CLL, clinicians use prognostic biomarkers and risk scoring systems to guide treatment decisions. In the last over 40 years, remarkable progress has been achieved through the identification of prognostic biomarkers capable of predicting survival and disease progression and reflecting the response to therapy. The conventional prognostic biomarkers and risk scoring systems have been validated in the CIT era. However, the prognostic value of the biomarkers and risk scoring systems in the context of novel agents needs further studies. In this review, we summarize prognostic biomarkers and risk scoring systems to identify the prognosis of CLL patients and discuss the possibility of using them in the era of novel agents.

### Prognostic biomarkers in CLL

The last three decades have generated a plethora of potential biomarkers. They range from serum markers to immunophenotypic markers, IGHV mutation status, chromosome aberrations, gene mutations, microRNAs and others. The application of these biomarkers in the context of novel agents have been studied in a series of studies. The recognized prognostic biomarkers in CLL are present in Table 1.

**Table 1:** Prognostic biomarkers in chronic lymphocytic leukemia

Category	Prognostic biomarkers
Serum markers	Thymidine kinase, beta2-microglobulin, lactic dehydrogenase, lymphocyte doubling time, autocrine interleukin-6, copper, free light chains, lipoprotein lipase, c-reaction protein, BAFF, TACI, APRIL, BCMA, EZH2
Immunophenotypic markers	CD38, ZAP70, CD49d, CD26, CD54, CD44, CD52, CD69, CD25, CD5, CD95, CD39, CD11c, CD36, CD150
IGHV mutation status	M-CLL, U-CLL
Chromosome aberrations	Del13q, del11q, tri12, del17p, del16q, del19p21, del10q23, total or partial trisomies of chromosomes 3, 8, 18, 19 and duplications in 2p24
Gene mutations	TP53, ATM, NOTCH1, BIRC3, MYD88, SF3B1, FBXWY, POT1, CHD2, RPS15, IKZF3, ZNF292, ZMYM3, ARID1A, PRPN11, COBLL1, LPL, ZAP70
Non-coding RNA and others	MiR-15a, miR-16-1, miR-155, miR-29a, miR-29b, miR-34a, miR-125a, miR-155, miR-181b, I-tRF-GlyCCC

### Serum markers

The serological test, which plays a crucial role in both diagnosis and evaluation of prognosis in CLL, is standard and inexpensive. Lymphocyte doubling time (LDT), serum beta2-microglobulin (s-β2M), serum thymidine kinase (s-TK) and lactic dehydrogenase (LDH) are the most common conventional serum markers in CLL and predict poor outcomes. LDT has been used as a prognostic parameter for more than 30 years. LDT ≤ 12 months predicts poor prognosis while LDT > 12 months correlates with a long treatment-free period and survive [10]. S-β2M level and s-TK level were reported as independent predictors of progression-free survival (PFS) of CLL more than 20 years ago [11]. S-β2M was widely used to improve risk stratification and retained independent prognostic value in several multiparameter scores [12–21]. Elevated s-TK level, which relates to shorter LDT and IGHV unmutated status, indicates the high risk of CLL patients and predicts disease progression [22]. LDH is an indicator of time to first treatment (TTFT) and associated with shorter PFS, overall survival (OS) and Richter’s transformation [23]. It is still of prognostic value in patients with trisomy 12 [24].

Besides, some other serum markers have also been suggested in recent years. The measurement of autocrine interleukin-6 (IL-6) could be a useful approach to predict clinical outcomes [25]. Higher serum copper level predicts a shorter time to start treatment and poor response to treatment. It is significantly associated with increased expressions of CD38 and ZAP70 [26]. Increased serum free light chains (sFLC) correlates with s-β2M, serum albumin, hemoglobin, abnormal LDH and regards to shorter time to treatment and OS [27]. Lipoprotein lipase (LPL) is an indicator of OS for CLL patients, while LPL mRNA expression, correlating with IGHV mutation status, also has a significant impact on survival [28]. Increased C-reaction protein (CRP) was approved as a predictor for shorter survival and associated with the development of second cancers [29]. The levels of serum B cell activator factor (BAFF), transmembrane activator and calcium-modulator and cyclophilin ligand interactor (TACI), the proliferation-inducing ligand APRIL, and B-cell maturation antigen (BCMA) were reported as novel predictors for OS [30, 31]. Enhancer of zeste homolog 2 (EZH2), a catalytic subunit of the initiation complex polycomb repressive complex 2 (PRC2), which was reported to mediate normal B cell and T cell lymphogenesis and modulate pathogenesis of lymphoid malignancies, is associated with more aggressive course in CLL [32, 33].

### Immunophenotypic markers

Based on the flow cytometry, CD38 and ZAP70 expression were validated to be accurate prognostic indicators of CLL [34]. Both of them can predict TTFT in the Binet 0 stage [35]. CD38 positivity, which is associated with other unfavorable factors such as higher s-β2M level and high-risk karyotypes, predicts the resistance of treatment, hepatomegaly and shorter survival [36, 37]. ZAP70 expression is associated with disease progression and is a predictor of Ritcher’s syndrome (RS) [38, 39]. CD38 positive identifies unmutated IGHV clones, while ZAP70 is a better indicator for IGHV mutation status [37]. However, another study investigated that it can predict treatment-free survival (TFS) and PFS in CLL patients but it was only in the absence of high-risk cytogenetic factors that is ZAP70 associated with IGHV mutation status [40]. CD49d expression is a strong independent factor of survival and treatment need. It is associated with the presence of lymphadenopathy at diagnosis and the development of lymphadenopathy during the course of the disease. CD49d drives disease progression and its expression pattern should also be considered to improve prognostic impact [41]. Besides, other CD markers may be associated with clinical symptoms and outcomes. CD26 expression is an independent indicator of time to treatment in CLL patients [42]. CD54, CD44, CD52, CD69, CD25, CD5, CD95, CD39, CD11c and CD36 expressions are indicators of poor outcomes, while CD150 predicts favorable course [36, 43].

### IGHV mutation status

The IGHV mutation status plays a pivotal role in the prognosis of CLL. According to the IGHV mutation status, patients can be divided into mutated CLL (M-CLL) patients and unmutated CLL (U-CLL) patients. Unmutated IGHV status, relating to shorter LDT and CD38 overexpression, is associated with a more aggressive course of CLL and predicts shorter TTFT in treatment-naïve patients, while M-CLL patients have better outcomes [44–47]. However, the correlation between U-CLL and CD38 expression was not found in another study [48]. Higher levels of CD47 are also related to U-CLL [49]. Compared to M-CLL, U-CLL is 4 times more likely to develop Richter syndrome (RS) [38]. In CLL patients with isolated del13q, U-CLL has shorter TTFT than M-CLL, whereas there was no significant difference in U-CLL and M-CLL in patients with tri12 [50]. According to BCR IG, patients can be assigned into subsets while the largest stereotyped subsets are #1, #2, #4 and #8. U-CLL subset #2 cases had shorter TTFT and TTNT than those in M-CLL, so the BCR IG subset #2 appeared as an independent prognostic factor [51]. Besides, a novel prognostic biomarker Fc receptor-like 2 (FCRL2) of low-risk CLL has prognostic value in CLL and predicts TTFT and OS [52]. The new prognostic biomarker was also able to further refine and extend prognosis in M-CLL patients.

### Chromosome aberrations

**Fig. 1:**
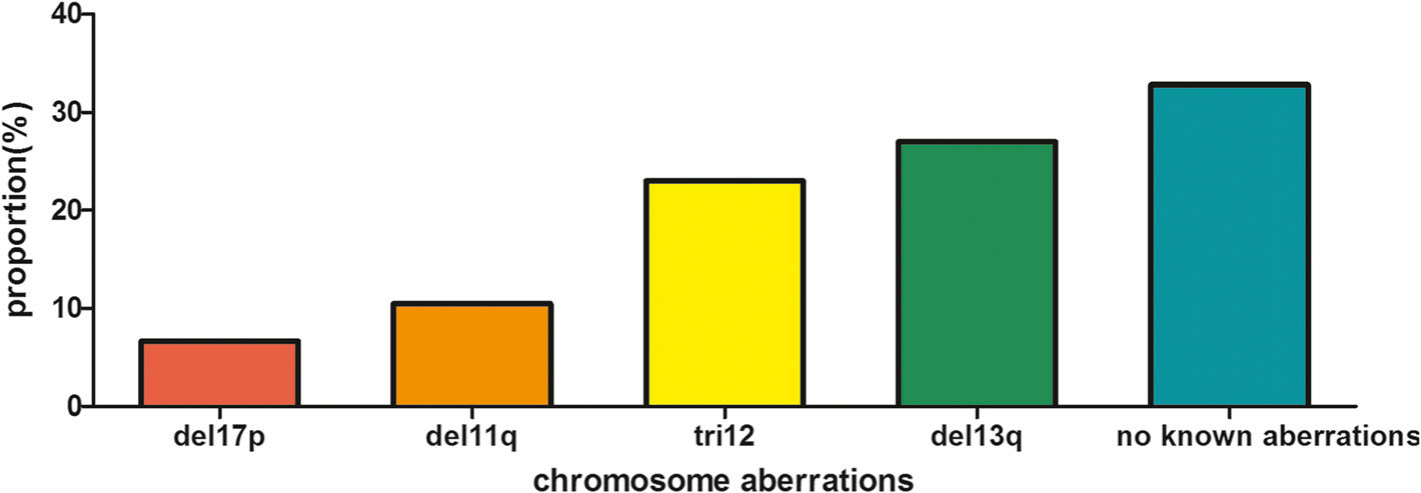
The percentage of common chromosome aberrations tested by fluorescence in situ hybridization

**Fig. 2:**
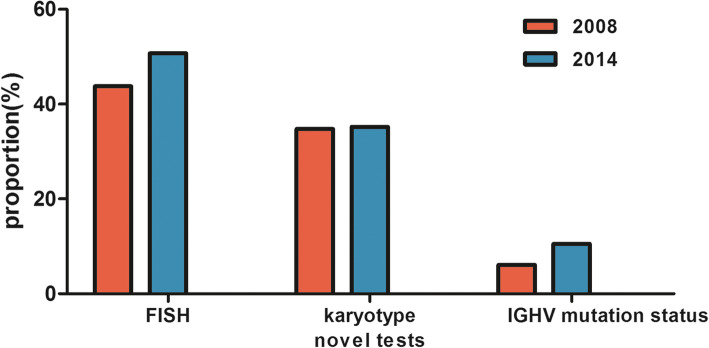
Comparison of fluorescence in situ hybridization (FISH), karyotype analysis and IGHV mutation test between 2008 and 2014

Fluorescence in situ hybridization (FISH), karyotype analysis and next-generation sequencing have been widely used in recent years in the diagnosis and risk stratification of CLL, making treatment decisions and designing clinical trials. Based on the data from SEER POC, Erlene K et al. proposed a real-world study and compared 1008 patients diagnosed in 2008 with 1367 patients diagnosed in 2014 [53], the most common chromosomal aberrations are deletions of the long arm of chromosome 13 (del13q), trisomy 12 (tri12), deletions of the long arm of chromosome 11 (del11q), and deletions of the short arm of chromosome 17 (del17p) (shown in Fig. 1). Besides, the performance of FISH test, karyotype analysis, and IGHV mutation test were all increased (shown in Fig. 2). The performance of these tests refines the risk stratification of CLL. Both chromosomal aberrations and gene mutations improve the prognostication of CLL patients.

Del13q, specifically involving band 13q14 on which miR-15a and miR-16a are located, seems to be the most frequently observed cytogenetic aberration in CLL and associated with a good prognosis [54, 55]. Patients with isolated del13q need treatment sooner and have a shorter OS if the cells with del13q ≥ 60% [56]. Both deletions of 13q14 and translocations with concomitant deletion at 13q14 can predict the unfavorable outcome of CLL [57]. A defective 5-methylcytosine (5-mCyt) status has association with a higher percentage of del13q, which suggests an aggressive process [58].

Tri12 is the second most frequent recurrent chromosomal aberrations in CLL and reported as an intermediate-risk cytogenetic aberration in newly diagnosed CLL patients [54]. A high proportion of cells with tri12 predicts worse outcomes [59]. CLL patients with tri12 present clinical and biological heterogeneity with the presence of additional genomic aberrations, such as trisomy 19 [60]. French Innovative Leukemia Organization (FILO) working group hosted a prospective study based on mutational and cytogenetic analysis of 188 CLL patients with tri12 [61]. The study showed that additional trisomies combined with tri12 were related to longer TTFT in Binet A stage patients and with a low risk of relapse [61].

Del11q is correlated with a worse prognosis. Patients with del11q have a shorter TFS but longer OS [62]. The lower frequency of del11q predicts the better outcome and the low frequency of gene mutations [63]. For the patients after allogeneic hematopoietic cell transplantation (allo-HCT), del11q, as well as del17q, can predict worse PFS and OS [64].

Del17p is found in 5–8% chemotherapy-naïve patients and associated with a dismal prognosis [2, 65]. As have been noted, del17p might influence the elimination of rituximab, which may explain the treatment resistance or rapid relapse [65]. The percentage of del17p cells has an impact on the prognosis of CLL patients, and the low percentage of del17p cells predicts a better TTFT [66]. However, another study revealed that neither del17p, mutated p53, nor complex karyotypes is associated with TTFT, which suggests that they have limited roles in early CLL patients but may take effect in relapsed disease [47]. Besides chromosome aberrations above, del6q, del9p21, del10q23, total or partial trisomies of chromosomes 3, 8, 18, 19, and duplications in 2p24 also have an impact on prognosis in CLL [67].

Complex karyotype (CK), defined by the presence of at least three cytogenetic abnormalities, is associated with poor outcomes [68]. Nevertheless, not all CKs are equivalent [69]. Panagiotis Baliakas et al. proposed a study that stratified CK patients into three subgroups based on whether they were carrying 3 or 4 or ≥ 5 abnormalities and defined them as low, intermediate and high CK, respectively [70]. According to this study, high-CK was associated with unfavorable outcomes, independently of the SHM and TP53 status.

### Gene mutations

In addition, gene mutations also play a pivotal role in CLL prognosis. The gene mutations examined by next-generation sequencing are associated with unmutated IGHV genes, CD 38 expression, and CK [71]. There have been 44 recurrently mutated genes and 11 recurrent somatic copy number variations identified in recent years [72].

NOTCH1 mutation, which is a predictor for advanced disease, is associated with tri12 [73]. The gene ATM, located on band 11q23, is associated with poor outcomes. Patients with del11q tend to harbor mutated ATM. Both del11q and mutated ATM may predict reduced survival [74]. BIRC3 mutation, also located on 11q and always coexisted with ATM deletion, erects a prognostic role in CLL patients [75]. The prominent tumor suppressor gene TP53 is located in the band 17p13. TP53 disruption relates to unfavorable prognosis in CLL patients [76]. The overexpression of a novel oncogene maternal embryonic leucine zipper kinase (MELK) predicts inferior survival in CLL and correlates with deletion of 17p13, as well as higher WBC count, advanced stage, elevated LDH, increased β2-M, unmutated IGHV status and positive ZAP70 [77].

Besides, MYD88, SF3B1, FBXWY, POT1, CHD2, RPS15, IKZF3, ZNF292, ZMYM3, ARID1A, and PTPN11 are also of the significant value of predicting the outcome of CLL patients [2]. NOTCH1 is the most frequently mutated gene, followed by XPO1, SF3B1, FBXW7, TP53, and MYD88 [78]. In treatment-naïve patients, mutated ATM, NOTCH1, and SF3B1 is correlated to shorter TTFT [47]. COBLL1, LPL, and ZAP70 gene expression is correlated to IGHV mutation status and is a predictor for OS and TTFT of CLL patients [79]. The number of mutations also has an impact on the prognosis. More than 2 mutations are independently associated with a shorter TTFT [71]. Genetic dynamics in untreated CLL patients suggests that monitoring variant allele frequency of a special gene panel may predict disease progression [80].

### MicroRNAs and others

MicroRNAs (miRNAs) are a group of small non-coding RNAs and play a crucial role in the regulation of gene expression. Human cancer is associated with miRNA expression, including CLL [81–83]. MiR-15a and miR-16-1, located on 13q14, were the earliest miRNAs used in the prognosis of CLL. They behave as tumor suppressors in CLL and are associated with TTFT [82]. More and more miRNAs were proved to have prognostic value in CLL. MiR-34a, targeting ZAP70 mRNA expression, is associated with the chemotherapy-refractory disease [84]. The downregulation of miR-34a and miR-125a upregulation was found to be associated with RS [85]. MiR-155 was reported as the most prevalent oncomiR in B-cell malignancies and revealed as a poor predictor for CLL, as well as an indicator to predict the therapy response [84, 86]. MiR-29a and miR-29b overexpression leads to aggressive CLL, as well as miR-155 [87]. MiR-129-2 methylation is associated with poor survival in CLL [88]. The expression of miRNAs also correlates with ethnicity. A study in the Chinese Uygur and Han populations revealed that the expression levels of miR-155, miR-29b, miR-181a, and miR-181b were associated with IGHV mutation and the low expression levels of miR-34a, miR-29b and miR-181b may be affected by p53 abnormality [89]. Besides miRNA, other non-coding RNAs were also estimated to predicts outcomes of CLL [90]. For example, I-tRF-GlyCCC, a fragment originating from tRNAs bearing the glycine anticodon CCC, was found unfavorable prognostic value in CLL patients [91].

All of the biomarkers above have prognostic value to evaluate the risk of disease progression and death. But only del 17p/ TP53 mutation has definitive predictive value related to the response of specific therapy. NOTCH1 mutation, associated with unfavorable response of anti-CD20 therapy, has potential predictive value to tailor clinical decision [92]. Other proposed predictive biomarkers were also reported, including IGHV mutation status, CD49d, CK and miRNA. (shown in Table 2).

**Table 2:** Clinical significance of predictive biomarkers in chronic lymphocytic leukemia

Predictive biomarkers	Clinical significance
Del 17p/ TP53 mutation	Predicts poor response to chemo-immunotherapy
NOTCH1 mutation	Predicts poor response to anti-CD20 therapy
CD49d	Inhibits cell trafficking in the setting of novel BCR target therapy
IGHV mutation status	Gives its potential for long-term remission in the use of BCR in younger, fit patients with M-IGHV
Complex karyotypes	Predict poor response to chemo-immunotherapy when complex karyotypes with major structural abnormalities.
MicroRNAs	MiR-34a: associates with chemotherapy-refractory disease MiR-155: predicts therapy response

### Risk staging systems in CLL

**Fig. 3:**
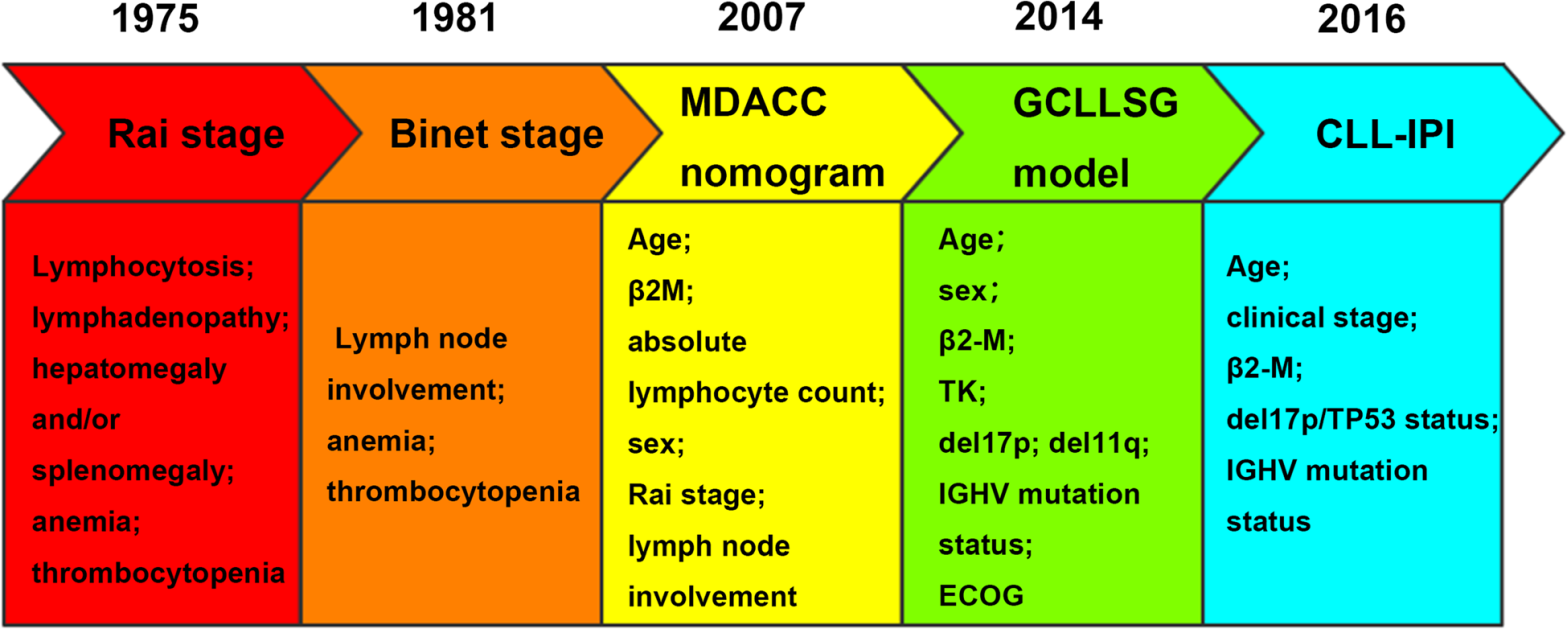
The risk factors of the classical prognostic models or staging systems. The Rai and Binet staging systems, MDACC nomogram, GCLLSG, CLL-IPI are the base of other prognostic models. It can be seen that the risk factors altered from the combination of clinical features and laboratory features to the combination of clinical and laboratory features with cytogenetic features

Based on the study of prognostic factors, the investigators integrated age, gender, performance status of patients and the prognostic biomarkers above to make risk stratification more precise and robust. For the purpose of identifying the risk groups, the classic prognostic staging systems, such as Rai and Binet staging systems, MDACC nomogram, GCLLSG model and CLL-IPI, were published. It can be seen that the risk factors altered from the combination of clinical features and laboratory features to the combination of clinical and laboratory features with cytogenetic features. (shown in Fig. 3) On the base of these models, more risk scoring systems were reported to assist in risk stratification. The detailed information about prognostic models can be seen in Table S1.

Rai and Binet staging systems, published in 1975 and 1981 separately, were the most widely used conventional staging systems in the clinical practice. The two staging systems based on the physical examination, including peripheral blood, lymph nodes, liver, spleen, and bone marrow [93, 94]. In comparison between the Rai stage and the Binet stage, it seems that the Rai stage is more accurate in patients with an excellent prognosis [95]. They used inexpensive and standard tools to identify 3 major prognostic subgroups. Furthermore, they are the basis of other prognostic models. However, the two staging systems fail to discriminate amongst patients at an early stage those who will experience an aggressive disease course. In addition, they do not incorporate the newly published biological characters, such as FISH and next-generation sequencing outcomes. Otherwise, the two staging systems have limited predictive power rewarding the response to therapy.

After Rai and Binet stage, MD Anderson Cancer Center (MDACC) group proposed a prognostic nomogram to predict OS for CLL patients, which combined age, β2M, absolute lymphocyte count, Rai stage and number of involved lymph node group in 2007 [12]. The nomogram can be used in early stage patients but the effect to therapy was not validated. Thus, Stefano Molica et al. modified the MDACC nomogram by changing cutoff to predict TTFT in Binet A stage patients in 2010 [13]. In addition, Pietro Bulian et al. integrated del17p and IGHV mutation status into the comprehensive model which includes Binet stage, β2M, age, and gender to estimate OS in 2012 [14]. The new model made up the deficiency that the MDACC nomogram did not include the biological and cytogenetic factors.

In 2014, GCLLSG published a new prognostic score. The new prognostic score incorporated sex, age, Eastern Cooperative Oncology Group (ECOG) status, del17p, del11q, IGHV mutation status, s-β2M and s-TK, which classified 4 risk categories [15]. The new prognostic score was validated in newly diagnosed CLL patients from the Mayo clinic [96]. It made up the deficiency of the Rai and Binet staging systems which lacked the new prognostic biomarkers. However, IGHV mutation status and s-TK level are not widely available. To reinforce the management of early stage CLL patients without s-TK level, Stefano Molica et al. proposed a modified version which not included s-TK assessment [16]. Indeed, it was also validated to predict the TTFT of CLL patients in the early stage with other 7 independent prognostic factors.

The CLL International Prognostic Index (CLL-IPI), the most widely used scoring system in clinical management, combines both clinical parameters and cytogenetic factors into a prognostic model in 2016. It contains 5 independent prognostic factors: TP53 status, IGHV mutational status, s-β2M concentration, clinical stage and age and identified 4 risk groups [97]. Compared with the nomogram proposed by the MDACC group, the CLL-IPI included cytogenetic abnormalities and was confirmed higher prognostic value in both OS and TTFT for newly diagnosed patients [12, 98, 99]. It predicts both PFS and OS and can be used in varieties of CIT approaches [100]. However, IGHV mutational status was only available in 10.1% CLL patients in 2014 in the United States [53]. Due to the correlation between LDT and IGHV mutational status, Deepesh P. Lad et al. created the modified CLL International Prognostic Index (CLL-LIPI), replacing IGHV unmutated status to LDT < 6 months and maintaining the original score of CLL-IPI [18]. It shows a comparable prognosis of OS with CLL-IPI. Compared with IGHV mutation status, LDT is more inexpensive and available. In the absence of IGHV mutational status, the CLL-LIPI still plays a crucial role in identifying the prognosis of CLL patients. Of note, the single-center study, which enrolled only 218 patients, can predict TTFT but not TTNT. In addition, CLL-IPI has a higher value in predicting the overall outcome [101]. To evaluate CLL-IPI in R/R CLL with novel therapy, Jacob D. Soumerai et al. found that the value of each prognostic indicator might be different compared with untreated CLL [19]. Thus, they modified cutoff for the clinical stage and assigned 1 point to each indicator. The modified score may improve the assessment of R/R CLL prognosis but is restricted to the CLL-IPI variables.

Based on the classic staging systems or prognostic models above, there have been other prognostic models reported in recent years. For patients with R/R CLL, Jacob D Soumerai et al. developed a prognostic risk score (BALL), which consisted of 4 factors, including s-β2M, LDH, hemoglobin and time from initiation of last therapy and separated patients into 3 subgroups [20]. This risk score reliably identifies treated patients with an increased risk of death and can be used to stratify patients in future clinical trials.

For patients after reduced-intensity conditioning allogeneic hematopoietic cell transplantation (RIC HCT), JR Brown et al. proposed a model to predict outcomes for patients undergoing reduced-intensity allogeneic stem cell transplantation in 2013 [102]. It incorporated remission status, LDH, comorbidity score, and lymphocyte count and separated patients into 4 subgroups. It can predict both PFS and OS for patients.

For patients with early stage, Manuela A. Hoechstetter et al. raised a prognostic model (CLL1-PM) for newly diagnosed CLL patients in Binet A stage by a multicenter, prospective CLL1 trial of the German CLL study group [21]. Del17p, unmutated IGHV, del11q, s-β2M > 3.5 mg/dL, LDT < 12 months and age > 60 years were identified as 6 independent factors and associated with OS and TTFT. These factors were integrated into CLL1-PM and separated patients into 4 risk subgroups. Although the new prognostic model has advantages to the management of newly diagnosed patients in the early stage, the outcomes of next-generation sequencing were not included.

There are other models that only incorporated two risk factors. Julio Delgado et al. built a model comprising IGHV mutation status and FISH cytogenetics (del11q and del17p) and separated patients into 3 risk groups [103]. It was validated in two independent cohorts and had similar discriminatory with CLL-IPI. Thus, the model may simplify the risk stratification in clinical management. Tamar Tadmor et al. proposed a new risk model based on heavy+light chains (HLC) and IgG subclasses to predict TTFT [104]. The model separated patients into 3 subgroups according to the numbers of risk factors and patients with 2 risk factors are of ultra-high-risk with a median TTFT of only 1.3 months. This study demonstrated the potential for the use of HLC and FLC immunoassays in future prognostication. Although all of the models above need to be further validated, they can improve the clinical management for CLL patients and help design clinical trials better.

However, the emergence of novel agents, such as Bruton tyrosine kinase (BTK) inhibitor ibrutinib, acalabrutinib and zanubrutinib, the phosphoinositide 3-kinase (PI3K) inhibitor idelalisib, the BCL-2 inhibitor venetovlax and other novel agents targeting different pathways, revolutionized the treatment and prognosis [105–107]. Targeting the promising biomarker EZH2, tazemetostat (EPZ-6438) shows antitumor activity in patients with refractory B-cell non-Hodgkin lymphoma [33, 108, 109]. Combination therapy with EZH2 inhibitors improves the efficacy of responses to signaling inhibitors [32]. In era of novel targeted agents, validations of the traditional prognostic parameters are warranted. Of note, many prognostic indicators validated in the era of CIT, including del17p/TP53 mutation, del11q, and unmutated IGHV status, are not of prognostic value in patients with relapsed/refractory CLL undergoing target therapy [110]. Adam S. Kittai et al. reviewed literature and concluded that high-risk features did not have the same impact on clinical outcomes in the era of the novel agent when compared with CIT, and only del17p/TP53 mutation and CD49d expression still predicted the inferior outcomes [111]. Patients with TP53 mutation undergoing ibrutinib ± rituximab have shorter PFS and OS, while patients undergoing idelalisib have shorter OS [112]. Del17p plays a significant role in predicting PFS in patients undergoing obinutuzumab + venetoclax [113]. In ibrutinib-treated patients, CD49d predicts reduced lymphocytosis and inferior nodal response and behaves as an independent predictor of shorter PFS [114]. The minimal residual disease (MRD) level is a prognosis indicator for PFS and OS of CLL patients with CIT [115]. Achieving MRD negativity may provide a more significant benefit for risk change, but the prognostic value of MRD in patients with novel agents needs more validated [116]. Indeed, the prognostic value of traditional biomarkers needs further study.

## Conclusion

In summary, there are a series of prognostic indicators and models can be used in the context of CIT. A better understanding of prognostic and predictive biomarkers helps to contribute to predicting survival and response to therapy in CLL. The classic scoring systems, such as the Rai and Binet staging systems, MDACC nomogram, GCLLSG model and CLL-IPI, incorporated them to improve risk stratification and guide treatment decisions. In the setting of novel therapy, current studies reported that only del 17p/TP53 mutation and CD49d expression still have prognostic value. The prognostic value of other biomarkers and models in the context of novel agents needs further studies.

## Supplementary information

**Additional file 1: Table S1**. Prognostic models or staging systems in chronic lymphocytic leukemia patients.

## Data Availability

Not applicable.

## Abbreviations

CLL: chronic lymphocytic leukemia
CIT: chemoimmunotherapy
CAR-T: chimeric antigen receptor T-cell
allo-SCT: allogeneic stem cell transplant
s-TK: serum thymidine kinase
PFS: progression-free survival
CLL-IPI: chronic lymphocytic leukemia international prognostic index
GCLLSG: German chronic lymphocyte leukemia study group
LDH: lactic dehydrogenase
TTFT: time to first treatment
OS: overall survival
tri12: trisome 12
LDT: lymphocyte doubling time
IL-6: interleukin-6
sFLC: serum free light chains
LPL: lipoprotein lipase
IGHV: immunoglobulin heavy chain variable region
CRP: C-reaction protein
RS: Ritcher’s syndrome
TFS: treatment-free surviaval
miRNA: microRNA
BAFF: B cell activator factor
TACI: transmembrane acivator and calcium-modulator and cyclophilin ligand interactor
BCMA: B-cell maturation antigen
EZH2: enhancer of zeste homolog 2
PRC2: polycomb repressive complex 2
FISH: fluorescence in situ hybridization
del13q: deletions of the long arm of chromosome 13
5-mCyt: 5-methylcytosine
del11q: deletions of the long arm of chromosome 11
allo-HCT: allogeneic hematopoietic cell transplantation
FILO: French Innovative Leukemia Oraganization
TTNT: time to next treatment
del17p: deletions of the short arm of chromosome 17
MELK: maternal embryonic leucine zipper kinase
CK: complex karyotype
MDACC: MD Anderson Cancer Center
ECOG: Eastern Cooperative Oncology Group
RIC HCT: reduced-intensity conditioning allogenetic hematopoietic cell transplantation
BTK: Bruton tyrosine kinase
PI3K: phosphoinositide 3-kinase
MRD: minimal residual disease
