# Supplementary material for: Recent progress of prognostic biomarkers and risk scoring systems in chronic lymphocytic leukemia
Source: Biomark Res. 2020 Sep 7;8:40. doi: 10.1186/s40364-020-00222-3 (PMC7487566; doi:10.1186/s40364-020-00222-3)
Supplement: Supplementary file 1 — Additional file 1: Table S1. Prognostic models or staging systems in chronic lymphocytic leukemia patients. [file 40364_2020_222_MOESM1_ESM.docx]

**Additional File**

**Table S1. Prognostic models or staging systems in chronic lymphocytic leukemia patients**

| **Prognostic models or staging systems** | **Year of publication** | **n** | **Varieties in models** | **Risk strategies** |
| --- | --- | --- | --- | --- |
| Rai stage | 1975 | 125 | Lymphocytosis;  lymphadenopathy;  hepatomegaly and/or splenomegaly;  anemia; thrombocytopenia | 5 stages (stage0-IV) |
| Binet stage | 1981 | 129 | Lymph node involvement;  anemia;  thrombocytopenia | 3 stages (stage A-C) |
| MDACC nomogram | 2007 | 1674 | Age;  sex;  Rai stage;  β2M;  absolute lymphocyte count;  lymph node involvement | 3 risk groups |
| Modified MDACC nomogram in Binet A stage | 2010 | 310 | Same as the MDACC nomogram | 3 risk groups |
| Modified MDACC nomogram | 2012 | 620 | Age;  sex;  Binet stage;  β2-M;  IGHV mutation status;  del17p | 3 risk groups |
| DFCI model | 2013 | 108 | Remission status;  LDH;  Comorbidity score;  lymphocyte count | 4 risk groups |
| GCLLSG model | 2014 | 1948 | age;  sex;  ECOG;  β2-M;  TK;  del17p;  del11q;  IGHV mutation status | 4 risk groups |
| Modified GCLLSG model | 2015 | 338 | age;  sex;  ECOG;  β2-M;  del17p;  del11q;  IGHV mutation status | - |
| CLL-IPI | 2016 | 3472 | Age;  clinical stage;  β2-M;  del17p/TP53 status;  IGHV mutation status | 4 risk groups |
| A prognostic model comprising only 2 biomarkers | 2017 | 524 | IGHV mutation status;  FISH cytogenetics (del17p and/or del11q) | 3 risk groups |
| Modified CLL-IPI in R/R CLL | 2019 | 897 | Age;  clinical stage;  β2-M;  del17p/TP53 status;  IGHV mutation status | 3 risk groups |
| A prognostic score for R/R patients | 2019 | 2475 | β2-M;  LDH;  hemoglobin;  time from initiation of last therapy | 3 risk groups |
| The prognostic score based on heavy chain immunoparesis and summated free light chains | 2019 | 122 | Heavy chain immuoparesis;  summated free light chains | 3 risk groups |
| CLL-LIPI | 2020 | 471 | Age;  Rai stage;  β2-M;  LDT  del17p; | 4 risk groups |
| CLL1-PM | 2020 | 539 | Age;  β2-M;  LDT;  IGHV mutations;  del17p;  del11q | 4 risk groups |
